# Left anterior descending artery dissection with retrograde aortic dissection during percutaneous coronary intervention: a case report

**DOI:** 10.3389/fsurg.2023.1236734

**Published:** 2023-08-15

**Authors:** Seha Ahn, Heejin Lee, Jung Suk Choi, Youngkyu Moon, In-Sub Kim, Si Young Choi, Joon Kyu Kang

**Affiliations:** Department of Thoracic & Cardiovascular Surgery, Eunpyeong St. Mary’s Hospital, College of Medicine, The Catholic University of Korea, Seoul, Republic of Korea

**Keywords:** LAD dissection, retrograde aortic dissection, percutaneous coronary intervention, catheter-induced coronary artery dissection, off-pump coronary artery bypass graft

## Abstract

Retrograde catheter-induced coronary artery dissection during percutaneous coronary intervention is an exceedingly rare occurrence, and the likelihood of it extending into the aorta is even more uncommon. Typically, surgical treatment involves aortic root replacement combined with coronary artery bypass grafting. However, in this particular case, a meticulous approach was employed. By carefully guiding wires into the true lumens and placing stents in the proximal left main and left anterior descending arteries, the immediate complications were averted by obstructing the retrograde flow in the false lumen. Subsequently, an off-pump coronary artery bypass was performed using the left internal mammary artery to the left anterior descending artery, without the need to manipulate the aorta. This approach resulted in a short operation time and the absence of any other complications.

## Introduction

Advances in cardiovascular interventional technology and stent materials are promoting an increase in the number of percutaneous coronary intervention (PCI) procedures and the number of patients undergoing complex, high risk PCI treatments. Among the catheter-induced coronary artery dissections (CICADs) that can occur during PCI, type A aortic dissection following the retrograde extension of a coronary artery dissection is one of the most life threatening and dramatic complications (1). In general, depending on the patient's condition, treatment typically requires emergency surgical procedures such as aortic root replacement accompanied by coronary artery bypass grafting. Additionally, critical care measures are implemented, including intubation, pacemaker stimulation to address bradycardia, infusion of inotropic drugs, utilization of an intra-aortic balloon pump, and potentially employing extracorporeal life support (2).

The elderly woman who experienced a mid-left anterior descending (LAD) arteries dissection during PCI underwent a successful rescue and repair procedure using a combination of two approaches. The procedure involved a PCI in the left main (LM) to mid-LAD arteries, followed by an emergency off-pump coronary artery bypass graft (OPCAB) utilizing the left internal mammary artery (LIMA) to LAD.

## Case presentation

**Figure 1:**
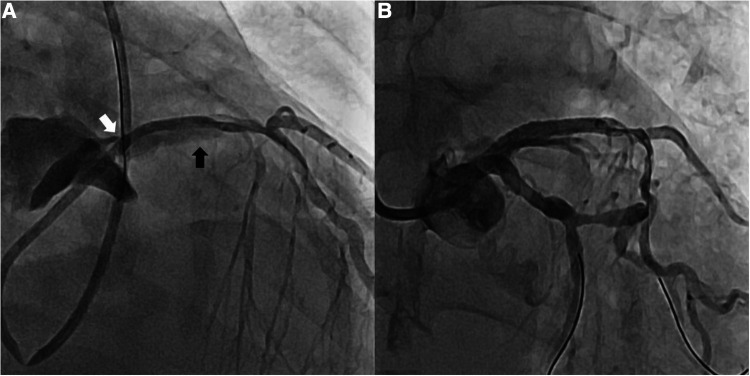
Coronary angiography. (**A**) Retrograde mid-LAD dissection (black arrow) and propagation to aortic dissection (white arrow) (**B**) Disappearance of mid-LAD dissection after PCI on LM to proximal LAD. LAD, left anterior descending artery; LM, left main coronary artery; PCI, percutaneous intervention.

**Figure 2:**
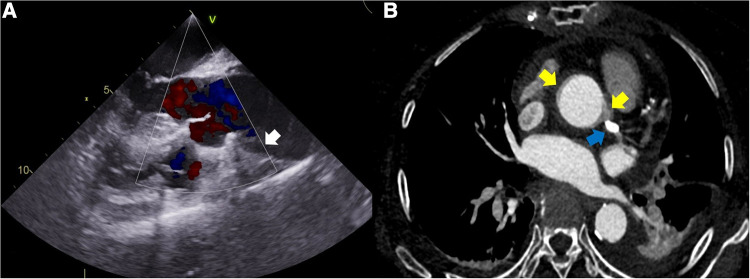
(**A**) Perioperative transesophageal echocardiography showing the flap of the proximal aortic dissection (white arrow). (**B**) Postoperative computed tomography showing a small remnant intramural hematoma (two yellow arrows) and the stent in the LAD (blue arrow). LAD, left anterior descending artery.

A 78-year-old woman was brought to our hospital with elevated cardiac enzymes. On admission, the electrocardiogram showed no ST elevation, but right bundle branch block; transthoracic echocardiography (TTE) showed moderate to severe hypokinesis at the anterior apex and the apical septal wall. Computed tomography (CT) showed neither aortic dissection nor pericardial effusion. With the diagnosis of non-ST elevation myocardial infarction, diagnostic coronary angiogram was performed and showed no stenosis or thrombus in LM, but diffuse, irregular, up to 85% stenosis from proximal to distal LAD and diffuse, irregular, up to 70% stenosis in the proximal to distal right coronary artery (RCA). When a mid-LAD dissection and retrograde aortic dissection appeared during PCI (Figure 1A), the PCI was carried out from LM to mid-LAD. However, once we confirmed that the balloon and stent would not pass the mid to distal LAD lesion, we proceeded with emergency OPCAB. Perioperative transesophageal echocardiography showed the flap of the proximal aortic dissection (Figure 2A). Median sternotomy was performed under general anesthesia, and there was moderate pericardial adhesion of unknown cause. Cardiopulmonary bypass (CPB) was considered unnecessary due to the absence of retrograde flow to the aorta in the operative field. Because the right and left saphenous veins were both <1 mm in diameter, saphenous vein to RCA bypass graft was not performed. LIMA to LAD OPCAB was performed, and an excellent result was obtained on the flow meter. Operation time was 89 min and anesthesia time was 125 min. The estimated blood loss was 700 ml. Postoperative TTE showed normal left ventricle global systolic function without definite regional wall motion abnormality. Postoperative CT revealed a small remnant intramural hematoma, and the LAD stent was visible (Figure 2B). The postoperative course was good and the patient was discharged from our hospital on postoperative day 9.

## Discussion and conclusions

CICADs are rare but can be severe complications that may arise during PCI (1). While minor dissections are typically easier to handle, dealing with more complex dissections can be quite challenging. The preferred treatment approach involves immediate stenting when the wire is positioned within the true lumen and stent implantation is feasible (3). Globally, the occurrence rate of aortic dissection during PCI is extremely low, measuring less than 0.1%. However, there is a scarcity of available data regarding the natural progression of this condition and its long-term outcomes (4). Rylski and his colleagues reported that immediate surgical treatment, including replacement of the ascending aorta and selective repair of the aortic root and arch, yielded acceptable results after iatrogenic type A aortic dissection, similar to those following naturally occurring aortic dissection (5). Verevkin and his colleagues have also noted that iatrogenic type A aortic dissection should be considered a surgical emergency because of the concern for extension of the dissection (1). However, Costa and his colleagues have recently reported that prompt percutaneous closure of the dissection point of entry was effective in managing iatrogenic LM and aortic root dissection (6). In a case report presented by Hung and his colleagues, they described an iatrogenic aortocoronary dissection that occurred during a routine PCI for stable angina. To address this complication, the team carefully guided wires through the true lumen and successfully placed a stent to seal the dissection flap, leading to the prevention of immediate complications and the resolution of a large mural hematoma in the mid-ascending aorta. Utilizing computed tomography aortography to guide their decisions, they opted for a conservative approach to manage the residual aortic dissection (7). In our case, the patient was of advanced age and we were attempting to manage a retrograde aortic dissection by PCI with stenting from LM to LAD. An emergency OPCAB (LIMA to LAD) was performed on the same day because of severe calcification of the coronary arteries, especially from mid- to distal LAD, and failure to pass a coronary wire into the true lumen for revascularization. The operation went well without any events, and the patient was discharged on postoperative day 9.

In conclusion, the most effective approach to address iatrogenic type A aortic dissection during PCI remains a topic of ongoing debate. The required treatments can encompass a combination of PCI, emergency surgery, and intensive care, which are tailored to the individual patient's condition. Our report presented a detailed case that showcased the successful integration of PCI and emergency OPCAB, aiming to enhance the quality of care and management for patients encountering iatrogenic type A aortic dissection.

## Data Availability

The original contributions presented in the study are included in the article/Supplementary Material, further inquiries can be directed to the corresponding author.
